# Parental genetic similarity and offspring performance in blue tits in relation to brood size manipulation

**DOI:** 10.1002/ece3.5367

**Published:** 2019-09-03

**Authors:** Aneta Arct, Szymon M. Drobniak, Samantha Mellinger, Lars Gustafsson, Mariusz Cichoń

**Affiliations:** ^1^ Institute of Environmental Sciences Jagiellonian University Kraków Poland; ^2^ Department of Animal Ecology/Ecology and Genetics, Evolutionary Biology Centre Uppsala University Uppsala Sweden

**Keywords:** birds, brood size manipulation, immune response, inbreeding, mate choice, microsatellite, passerine, relatedness

## Abstract

In birds, as in many other taxa, higher genetic similarity of mates has long been known to reduce offspring fitness. To date, the majority of avian studies have focused on examination whether the genetic similarity of social mates predicts hatching success. Yet, increased genetic similarity of mates may also reduce offspring fitness during later life stages, including the nestling period and beyond. Here, we investigated whether parental genetic similarity influences offspring performance using data from free‐living blue tits (*Cyanistes caeruleus*) collected across three breeding seasons. Additionally, we tested whether brood size manipulation affects the magnitude and direction of the relationship between genetic similarity of mates and offspring performance. Sixteen microsatellite markers were used to measure genetic similarity between biological parents. We found that the genetic similarity of parents negatively affects offspring immune response and this effect was independent of the experimental brood size manipulation.

## Graphical abstract

This is one of a few studies on natural populations of birds showing negative effects of parental genetic similarity on offspring performance. Specifically, we found that a higher genetic similarity of parents negatively affects offspring immune response to a novel antigen—phytohaemagglutinin.

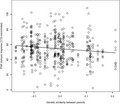

## 1 INTRODUCTION

Parental genetic similarity often reduces offspring fitness, probably because homozygosity leads to the expression of recessive deleterious alleles (Bensch, Hasselquist, & Schantz, 1994; Billing et al., 2012; Charlesworth & Charlesworth, 1987; Keller & Waller, 2002; Kempenaers, Adriaensen, Noordwijk, & Dhondt, 1996). There is accumulating evidence that reproductive success of mates becomes lower as their genetic similarity increases (reviewed in Keller & Waller, 2002; Spottiswoode & Møller, 2004; Amos et al., 2001; Wright, Tregenza, & Hosken, 2008). Specifically, in birds elevated parental genetic similarity is related to decreased hatchability (reviewed in Spottiswoode & Møller, 2004). However, little is known about effects of increased parental genetic similarity on posthatching development. The results of existing studies are equivocal. For example, studies on passerine birds found no effects of genetic similarity on fledgling survival (Kempenaers et al., 1996; Kleven, Jacobsen, Robertson, & Lifjeld, 2005; Krokene & Lifjeld, 2000; Schmoll et al., 2005). Similarly, in the house sparrow (*Passer domesticus*) there was no relationship between the parental genetic similarity and nestling body weight or immunocompetence (Edly‐Wright, Schwagmeyer, Parker, & Mock, 2007). In contrast, Freeman‐Gallant, Wheelwright, Meiklejohn, and Sollecito (2006) showed that the fledging weight and growth rates of sons decreased substantially with increasing genetic similarity of the parent mates, but only in one of the two studied breeding seasons. Moreover, in great tits (*Parus major*) parental genetic similarity reduced fledging success, although only late in the season, when environmental conditions are likely to be harsh (van de Casteele, Galbusera, Schenck, & Matthysen, 2003).

The available evidence, therefore, suggests that the relationship between genetic similarity and offspring fitness is detected and/or more pronounced under harsher environmental conditions and may be nonsignificant under favorable conditions. Indeed, Keller, Grant, Grant, and Petren (2002) found negative effects of mating between close relatives in Darwin's finches only under nutritional constraints.

Furthermore, there is evidence that environmental conditions may strengthen the relationship between heterozygosity and fitness, with stronger correlations arising under poor environmental conditions (Ferrer, García‐Navas, Sanz, & Ortego, 2016; Forcada & Hoffman, 2014; Voegeli, Saladin, Wegmann, & Richner, 2012). However, to our knowledge no experimental research has addressed this question.

Here, we investigate the relationships between genetic similarity of parents and offspring performance in the blue tit (*Cyanistes caeruleus*). Specifically, we investigated whether genetic similarity within the pair influences offspring quality in terms of body weight, tarsus length, and immunocompetence. We expected to see negative effects of parental genetic similarity on these three measured traits. If the relationship between genetic similarity of mates and offspring quality and its magnitude depend on environmental conditions, one can expect a significant interaction between parental genetic similarity and environmental conditions. We therefore manipulated the brood size to alter conditions of nestling growth, and we expected to see a negative relationship between genetic similarity of mates and nestling quality that will be particularly pronounced among offspring from experimentally enlarged broods.

## 2 METHODS

### 2.1 Study site and experimental procedure

Our study was carried out on Gotland, Sweden (57°03′N, 18°17′E), during April–June, from 2009 to 2011. Adult birds were caught while feeding nestlings (May–June), using nest‐box traps or mist nets. Birds were bled by brachial venepuncture for genetic analyses. All nestlings were weighed on days 2 and 14 prior to the blood sampling and on days 11 through 12 as a part of the immune response assay. Additionally, on day 14 after hatching, nestlings were measured for tarsus length and ringed. To assess nestling T‐cell‐mediated immune response, on day 11 posthatching nestlings were injected with a nonpathogenic antigen, phytohemagglutinin (PHA) into their right wing web, and 24 hr later, the thickness of the wing web was measured with a pressure‐sensitive calliper (see details in Drobniak et al. (2010) and Arct, Drobniak, Podmokła, Gustafson, and Cichoń (2013), Arct et al. (2017)). The measurements were taken by a single person and were highly repeatable (*r* = 0.92, *F*_461,924_ = 36.1, *p* < 0.0001). The mean value of the three repeats was used in further analyses.

Here, we analyzed data on blue tits subjected to a brood size manipulation treatment, which is known to alter conditions of nestling growth and has a negative effect on various nestling characteristics (Cichoń & Dubiec, 2005; Neuenschwander, Brinkhof, Kolliker, & Richner, 2003). The following treatment description takes into account the cross‐fostering experiment that was performed on all nests by exchanging halves of broods between nests and performed in another study (for details see Drobniak et al., 2010). Half of the nestlings were exchanged between control and experimental nests. Briefly, on the second day after hatching, we matched pairs of broods according to the same hatching date and similar brood size (±1 chick) (in year 2009:16 pairs, 2010:15 pairs and 2011:19 pairs of broods). One randomly chosen brood in each pair was subjected to brood size manipulation—enlargement by three nestlings coming from donor nests (ca. 30% increase in brood size), while the second brood was left unmanipulated, constituting the control group. Donor nests and donor nestlings were not included in further analyses. The birds were caught and manipulated under a ringing license from the Swedish Ringing Office (Stockholm Museum of Natural History), in accordance with the Swedish guidelines for work on natural populations.

### 2.2 Genetic analysis

Sexing of nestlings was performed by using the P2–P8 primers (Griffiths, Double, Orr, & Dawson, 1998) that amplify the sex‐specific CHD locus. Parentage was assigned by genotyping adults and chicks at 5–15 microsatellite loci, as described in the study by Arct et al. (2013) and Arct et al. (2017). In total, 30 out of 72 broods contained at least one extra‐pair young (EPY) and 55 out of 716 offspring were identified as EPY. We genotyped all adult birds (*N* = 238, 143 males and 140 females) using a panel of sixteen autosomal microsatellite markers: Ase18; PmaTGA45; PmaGAn40; PmaGAn27; PCA7; PCA4; PCA9; Pocc6; PMAC25; Mcyl4; PK12; Cdi31; PCA3; Pma303; Pocc1; and Pca8. The PCRs were performed with the Qiagen multiplex PCR kit (Qiagen AG, Hombrechtikon, Switzerland) as described in the study of Olano‐Marin et al. (2010). In our study, we used only neutral microsatellite markers (sensu Olano‐Marin, Mueller, & Kempenaers, 2011a, 2011b). We calculated the relatedness within a breeding pair using the R package DEMERELATE (Kraemer & Gerlach, 2013) and used the Wang estimator (2002) as a measure of genetic similarity of the pair members. This estimator ranges from −1 to 1, with negative values indicating that individuals share fewer alleles than average (Thuman & Griffith, 2005). We estimated the heterozygosity–heterozygosity correlations (HHC, Balloux, Amos, and Coulson (2004)) coefficient (*r*) and the 95% confidence intervals using the inbreed R package for R (Stoffel et al., 2016). We also calculated the parameter *g*_2_ as a measure of identity disequilibrium. There was no indication for inbreeding in our population: HHC and *g*_2_ for the markers were not significantly different from zero (i.e., 95% quantiles crossed zero) [all markers: *r*_HHC_ = 0.00, 95% CI = −0.098 to 0.094 (1,000 randomizations); *g*_2_ = 0.0007, 95% CI = −0.002 to 0.0009, *p* = 0.73 (based on 100 permutations)].

### 2.3 Statistical analysis

We used a linear mixed model (LMM) in R (R Development Core Team, 2014) with the add‐on R package lme4 (Bates, Maechler, Bolker, & Walker, 2014) to test for the effects of genetic similarity of biological parents on body mass on day 14, tarsus length, and the T‐cell‐mediated immune response to PHA. All the models included offspring sex and experimental treatment as fixed categorical variables. The genetic similarity between biological partners was entered as a covariate. Year of study, box of origin, and box of rearing were included as random factors. During data analysis, we first introduced a random effect of “pair ID” to control for the fact that nests were paired in cross‐fostering blocks according to hatching date and brood size. The variance of this parameter was virtually 0, and therefore, the term was culled as redundant, allowing model simplification. We tested only one interaction (parental genetic similarity × treatment), because we had clear a priori predictions. The interaction between parental genetic similarity and experimental treatment turned out nonsignificant (*p* > 0.05) for all the measured traits and was thus deleted from the final model (Table S1), which allow us to interpreted main results (e.g., Schielzeth, 2010).

In the analysis of the T‐cell‐mediated immune response, we used the body mass on day 12 (when the immune response was measured) as a covariate. The data met the assumptions of a linear mixed model (which we judged visually using model residuals). We used the “MuMIn” package in R to estimate the coefficient *R* ^2^ from the mixed models (Bartoń, 2009; Nakagawa & Schielzeth, 2013). Sample sizes differed between the analyses of body mass/ tarsus length on day 14 and analysis of immunocompetence, as measurements of the PHA immune response were not available for all the nestlings. In the final analyses, we removed extra‐pair nestlings, thus eliminating the potentially confounding effect of an extra‐pair sire genetic contribution to nestling performance. Inclusion of these extra‐pair young in analyses did not change the results (Table S2). We confirm that the total number of excluded observations and the reasons for making these exclusions have been reported in the Method section.

## 3 RESULTS

The interaction between parental genetic similarity and experimental treatment appeared nonsignificant for all measured traits (Table S1) and was thus deleted from the final model.

**Figure 1:**
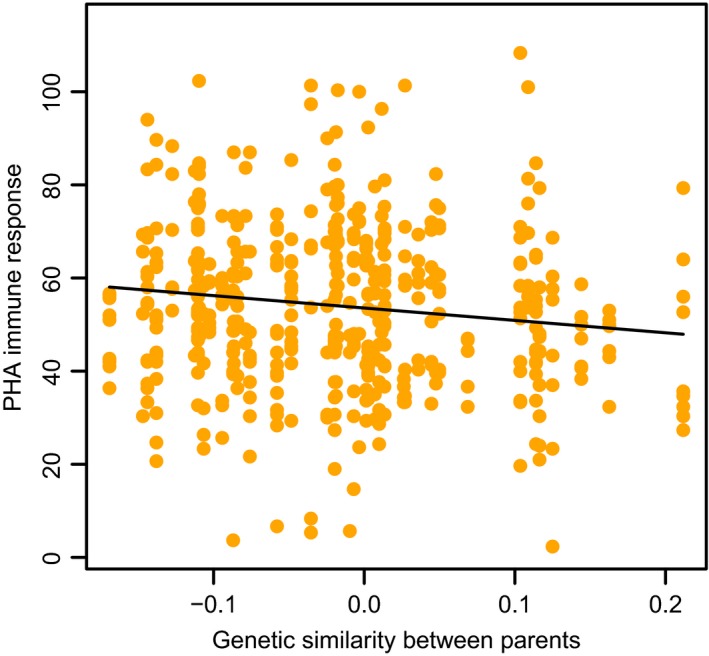
Nestling T‐cell‐mediated immune response to PHA in relation to parental genetic similarity. The graph shows the best‐fit regression line for the mean of the width variable. See the Methods section for details on statistics

We found a significant negative correlation between T‐cell‐mediated immune response to PHA and parental genetic similarity (Table 1, Figure 1). There were no effects of parental genetic similarity on body mass and tarsus length on day 14. Experimental treatment significantly affected the body mass of nestlings (means ± *SD*: control nests 11.25 ± 0.86 g; experimental nests 10.84 ± 1.14 g (Table 1) but had no significant effect on PHA immune response and tarsus length.

**Table 1:** Linear mixed model analyses (LMMs) of the body mass on day 14 (g), tarsus length (mm), and T‐cell‐mediated immune response to PHA

Body mass on day 14, N = 377					
Fixed effects	Estimate	*SE*	*df*	*t*	*p*
Intercept	11.03	0.24	2.59	46.87	**<0.001**
Parental genetic similarity	0.88	1.02	49.83	0.87	0.389
Experimental treatment	−0.37	0.13	46.8	−2.8	**0.007**
Sex	0.52	0.09	340.07	5.71	**<0.001**

Random effects	Variance	*SD*
Nest of rearing	0.167	0.41
Nest of origin	0.218	0.47
Year of study	0.108	0.33
Residual	0.67	0.82
*R* ^2^(m)	8.50%	
*R* ^2^(c)	47.31%	

Tarsus length on day 14, N = 377					
Fixed effects	Estimate	*SE*	*df*	*t*	*p*
Intercept	16.31	0.16	2.68	101.33	**<0.001**
Parental genetic similarity	0.6	0.64	46.81	0.937	0.354
Experimental treatment	−0.1	0.08	44.54	−1.223	0.228
Sex	0.36	0.05	332.35	6.81	**<0.001**

Random effects	Variance	*SD*
Nest of rearing	0.076	0.28
Nest of origin	0.086	0.29
Year of study	0.055	0.23
Residual	0.22	0.47
*R* ^2^(m)	8.51%	
*R* ^2^(c)	53.93%	

PHA immune response, N = 351					
Fixed effects	Estimate	*SE*	*df*	*t*	*p*
Intercept	12.56	9.19	194.68	1.37	0.173
Parental genetic similarity	−26.73	11.85	38.34	−2.26	**0.030**
Experimental treatment	−2.44	2.01	53.36	−1.22	0.229
Sex	−0.64	1.74	343.4	−0.36	0.715
Body mass on day 12	3.97	0.85	193.69	4.65	**<0.001**

Random effects	Variance	*SD*
Nest of rearing	12.02	3.47
Nest of origin	15.93	3.99
Year of study	0.00	0.00
Residual	231.17	15.20
*R* ^2^(m)	11.39%	
*R* ^2^(c)	20.89%	

Nest of rearing, nest of origin, and the year of study were included as higher‐level random effects. Parental genetic similarity was entered as a covariate; experimental treatment (experimental nests/control nests) and offspring sex (female/male) were defined as fixed factors. In the analysis of the T‐cell‐mediated immune response, we used the body mass on day 12 (when the immune response was measured) as a covariate. We present two types of *R* ^2^—marginal *R* ^2^(m) and conditional *R* ^2^(c) for both LMMs.

Bold indicates significant effects (*p* > 0.05).

## 4 DISCUSSION

In this study, we investigated the effect of brood size manipulation treatment on the relationship between genetic similarity and offspring performance. To our knowledge, the effect of environmental condition on the relationship between parental genetic similarity and offspring performance has never been experimentally investigated. However, we failed to find any evidence that the brood size manipulation experiment affects the relationship between genetic similarity and offspring performance. There is accumulating evidence that brood size manipulation has an effect on nestling immunocompetence and body condition (Horak, Tegelmann, Ots, & Moller, 1999; Sanz & Tinbergen, 1999). It is probable that brood enlargement creates a competitive environment for the offspring. Here, we showed that enlargement of the brood size affected nestling body mass on day 14. However, in the case of PHA immune response and tarsus length, we observed only a trend that offspring from experimental nests had lower immunocompetence and shorter tarsi. Such discrepancy may be a result of a relatively small sample size. This obviously reduces the power to find a significant interaction between experimental treatment and parental genetic similarity. Our previous studies on blue tits also did not support the idea that heterozygosity–fitness correlations become stronger under stressful conditions than under optimal conditions (Arct et al., 2017). This does not, however, exclude the possibility that other environmental factors, such as parasite prevalence, food availability, or harsh weather conditions, might strengthen the association between parental genetic similarity and offspring fitness.

Here, we showed that genetic similarity between pair members had a significant effect on offspring immunocompetence in blue tits. Specifically, in line with our prediction, we found a negative relationship between parental genetic similarity and offspring PHA immune response (Figure 1). Similar to the previous study on great tits (*P. major*) (van de Casteele et al., 2003), we showed that the effect of parental genetic similarity on offspring fitness‐related traits is not limited to embryonic stage but may also be important at later life stages. The ability to mount a strong immune response to pathogens was found to play an important role in determining individual survival prospects (Cichoń & Dubiec, 2005). Indeed, immune response was positively related to the probability of survival in nestlings (Cichoń & Dubiec, 2005) as well as adult birds (Gonzalez et al., 1999; Saino, Calza, & Møller, 1997), and the genetic variability in immune response to pathogens in natural populations is well documented (Cichoń, Sendecka, & Gustafsson, 2006; Drobniak et al., 2010; Saino et al., 1997). Thus, our study suggests that high parental genetic similarity may have potentially detrimental fitness consequences in blue tits. Previous studies on house sparrows (*P. domesticus*) (Edly‐Wright et al., 2007) failed to find a relationship between parental genetic similarity and offspring PHA immune response. It is possible that Edly‐Wright et al. (2007) did not detect such a relationship because in their genetic similarity assessment they used band sharing coefficients of DNA fingerprints. Griffith and Montgomerie (2003) raised doubts as to whether band sharing coefficients based on multi‐locus DNA fingerprints allow reliable evaluation of the genetic similarity between mates, because coefficients for different pairs of individuals of given relatedness show considerable random variation.

In our study, the negative relationship between parental genetic similarity and offspring fitness‐related traits was visible only in the immune response. Similarly, in our previous study on blue tits we found that the immune response of extra‐pair young to phytohemagglutinin was stronger than that of within‐pair half‐sibs, but at the same time the superior quality of EPY was not confirmed in terms of body mass and tarsus length. This could theoretically be due to a causal relationship between the individual level of genetic diversity and the PHA immune response (Fossøy, Johnsen, & Lifjeld, 2008). However, a correlation between the PHA immune response and individual level of heterozygosity was not supported in our previous studies on the same population of blue tits (Arct et al., 2017). In contrast, we found a positive relationship between individual heterozygosity and body mass of female nestlings 14 days posthatching. Thus, the observed relationship between parental genetic similarity and offspring immunocompetence may potentially be explained not only through genetic effects but also by maternal effects. Indeed, there is evidence that females may adjust their reproductive investment in response to the genetic similarity of their partners (Arct, Rutkowska, Martyka, Drobniak, & Cichoń, 2010). For example, differences in offspring performance may result from nongenetic maternal effects (Martyka, Rutkowska, & Cichoń, 2011). However, from our correlational study we cannot draw conclusions about which mechanism could explain the observed relationship between parental genetic similarity and offspring immunocompetence. It cannot be excluded that both genetic and maternal effects might interact together in this case.

In conclusion, this is one of a few studies on natural populations of birds showing negative effects of genetic similarity between pair members on offspring performance. More importantly, our data indicate that the effects of parental genetic similarity on offspring performance‐related traits are not limited to embryonic stage but are also important during later life stages. However, we failed to find any evidence that experimentally altered environmental conditions affect the relationship between parental genetic similarity and offspring fitness. Detailed studies are still needed to further test under which environmental conditions we may expect the relationship between the genetic similarity of mates and offspring fitness to vary.

## CONFLICT OF INTEREST

None declared.

## AUTHOR CONTRIBUTIONS

AA—contributed to the design of the work, genetic and statistical analysis, and interpretation of data, and wrote the manuscript; SMD—verified the analytical methods, designed the figures, and interpreted the data; SM—involved in genetic analysis; LG—supervised the long‐term study and worked on the manuscript; MC—contributed to the design and implementation of the research, to the analysis of the results and to the writing of the manuscript.

## Data Availability

All data and microsatellite genotypes are available through the Dryad Digital Repository: https://doi.org/10.5061/dryad.v6r0758.
