# Supplementary material for: Parental genetic similarity and offspring performance in blue tits in relation to brood size manipulation
Source: Ecol Evol. 2019 Sep 3;9(18):10085–91. doi: 10.1002/ece3.5367 (PMC6787802; doi:10.1002/ece3.5367)
Supplement: Supplementary file 1 [file ECE3-9-10085-s001.docx]

Table 1. Linear mixed-model analyses of the body mass on day 14 (g), tarsus length (mm) and T-cell mediated immune response to PHA. Nest of rearing, nest of origin and the year of study, were included as higher level random effects (results not shown). Parental genetic similarity was entered as a covariate, experimental treatment (experimental nests, control nests), offspring sex (female/male) were defined as fixed factors. In the analysis of the T-cell-mediated immune response we used the body mass on day 12 (when the immune response was measured) as a covariate.

| ***Body mass on day 14, N=377*** |  |  |  |  |  |
| --- | --- | --- | --- | --- | --- |
| **Fixed effects** | **Estimate** | **SE** | **df** | **t** | ***P*** |
| Intercept | 11.05 | 0.23 | 2.65 | 47.11 | **<0.001** |
| Parental genetic similarity | 1.53 | 1.21 | 81.76 | 1.26 | 0.211 |
| Experimental treatment | -0.41 | 0.14 | 44.66 | -2.92 | 0.005 |
| Sex | 0.53 | 0.09 | 339.89 | 5.75 | **<0.001** |
| Parental genetic similarity x Experimental treatment | -1.29 | 1.31 | 91.1 | -0.98 | 0.330 |
|  |  |  |  |  |  |
| ***Tarsus length on day 14, N=377*** |  |  |  |  |  |
| **Fixed effects** | **Estimate** | **SE** | **df** | **t** | **P** |
| Intercept | 16.32 | 0.16 | 2.74 | 100.86 | **<0.001** |
| Parental genetic similarity | 1.31 | 0.76 | 78.17 | 1.73 | 0.088 |
| Experimental treatment | -0.14 | 0.09 | 44.12 | -1.59 | 0.118 |
| Sex | 0.36 | 0.05 | 332.3 | 6.92 | **<0.001** |
| Parental genetic similarity x Experimental treatment | -1.42 | 0.8 | 104.25 | -1.77 | 0.080 |
|  |  |  |  |  |  |
| ***PHA immune response, N=351*** |  |  |  |  |  |
| **Fixed effects** | **Estimate** | **SE** | **df** | **t** | ***P*** |
| Intercept | 12.62 | 9.1 | 185.92 | 1.39 | 0.167 |
| Parental genetic similarity | -41.13 | 15.32 | 65.78 | -2.68 | **<0.001** |
| Experimental treatment | -1.82 | 1.99 | 41.66 | -0.92 | 0.365 |
| Sex | -0.67 | 1.74 | 341.3 | -0.39 | 0.700 |
| Body mass on day 12 | 3.93 | 0.85 | 186.47 | 4.65 | **<0.001** |
| Parental genetic similarity x Experimental treatment | 28.08 | 19.45 | 56.18 | 1.44 | 0.154 |
|  |  |  |  |  |  |

Table 2. Linear mixed-model analyses of the body mass on day 14 (g), tarsus length (mm) and T-cell mediated immune response to PHA. Nest of rearing, nest of origin and the year of study, were included as higher level random effects (results not shown). Parental genetic similarity was entered as a covariate, experimental treatment (experimental nests/control nests), offspring sex (female/male) and paternity (EPY-extra pair young/ WPY- within pair young) were defined as fixed factors. In the analysis of the T-cell-mediated immune response we used the body mass on day 12 (when the immune response was measured) as a covariate.

| ***Body mass on day 14, N=425*** | |  |  |  |  |
| --- | --- | --- | --- | --- | --- |
|  |  |  |  |  |  |
| **Fixed effects** | **Estimate** | **SE** | **df** | **t** | ***P*** |
| Intercept | 11.01 | 0.23 | 2.63 | 48.40 | **<0.001** |
| Parental genetic similarity | 0.86 | 1.04 | 52.06 | 0.82 | 0.415 |
| Experimental treatment | -0.34 | 0.14 | 49.31 | -2.43 | **0.019** |
| Sex | 0.54 | 0.10 | 388.95 | 5.64 | **<0.001** |
| Paternity | -0.35 | 0.15 | 400.69 | -2.27 | **0.024** |
|  | |  |  |  |  |
| ***Tarsus length on day 14, N=425*** |  |  |  |  |  |
| **Fixed effects** | **Estimate** | **SE** | **df** | **t** | ***P*** |
| Intercept | 16.31 | 0.16 | 2.63 | 101.60 | **<0.001** |
| Parental genetic similarity | 0.45 | 0.61 | 49.27 | 0.74 | 0.464 |
| Experimental treatment | -0.13 | 0.08 | 40.34 | -1.57 | 0.124 |
| Sex | 0.38 | 0.05 | 380.19 | 7.55 | **<0.001** |
| Paternity | 0.02 | 0.08 | 391.38 | 0.25 | 0.805 |
|  |  |  |  |  |  |
| ***PHA immune response, N=396*** | |  |  |  |  |
| **Fixed effects** | **Estimate** | **SE** | **df** | **t** | ***P*** |
| Intercept | 0.7 | 0.91 | 167.48 | 0.77 | 0.441 |
| Parental genetic similarity | -2.72 | 1.13 | 36.47 | -2.40 | **0.022** |
| Experimental treatment | -0.11 | 0.19 | 51.09 | -0.57 | 0.573 |
| Sex | -0.09 | 0.16 | 385.81 | -0.56 | 0.576 |
| Paternity | 0.38 | 0.25 | 388.46 | 1.50 | 0.133 |
| Body mass on day 12 | 0.44 | 0.08 | 220.51 | 5.32 | **<0.001** |
